# Experimental Investigation on Micro-Groove Manufacturing of Ti-6Al-4V Alloy by Using Ultrasonic Elliptical Vibration Assisted Cutting

**DOI:** 10.3390/ma12193086

**Published:** 2019-09-21

**Authors:** Rongkai Tan, Xuesen Zhao, Tao Sun, Xicong Zou, Zhenjiang Hu

**Affiliations:** 1 Center for Precision Engineering, Harbin Institute of Technology, Harbin 150001, China; tanrongkai17@gmail.com (R.T.); zhaoxuesen@hit.edu.cn (X.Z.); lyhoo@hit.edu.cn (Z.H.); 2 School of Mechatronics Engineering, Heilongjiang University, Harbin 150080, China; zouxicong@hlju.edu.cn

**Keywords:** micro-groove, titanium alloy, surface integrity, material swelling and springback, ultrasonic elliptical vibration assisted cutting

## Abstract

The micro-groove structure on the planar surface has been widely used in the tribology field for improving the lubrication performance, thereby reducing the friction coefficient and wear. However, in the conventional cutting (CC) process, the high-quality, high-precision machining of the micro-groove on titanium alloy has always been a challenge, because considerable problems including poor surface integrity and a high level of the material swelling and springback remain unresolved. In this study, the ultrasonic elliptical vibration assisted cutting (UEVC) technology was employed, which aimed to minimize the level of the material swelling and springback and improve the machining quality. A series of comparative investigations on the surface defect, surface roughness, and material swelling and springback under the CC and UEVC processes were performed. The experimental results certified that the material swelling and springback significantly reduced and the surface integrity obviously improved in the UEVC process in comparison to that in the CC process. Furthermore, for all the predetermined depths of the cut, when the TSR (the ratio of the nominal cutting speed to the peak horizontal vibration speed) was equal to one of twenty four or one of forty eight, the accuracy of the machined micro-groove depth, width and the profile radius reached satisfactorily to 98%, and the roughness values were approximately 0.1 μm. The experimental results demonstrate that the UEVC technology is a feasible method for the high-quality and high-precision processing of the micro-groove on Ti-6Al-4V alloy.

## 1. Introduction

Titanium alloys have been increasingly used in aerospace, aviation, shipbuilding and biomedical fields because of their excellent properties such as high yield stress, high toughness, high strength to weight ratio, high creep and corrosion resistivity and good biocompatibility [1]. However, the surface hardness of titanium alloy is not usually high (approximately 30 HRC), which leads to a poor wear resistance of the titanium alloy part [2,3]. In practical application, the failure of titanium alloy part is often caused by its poor wear resistance [4,5]. Therefore, the studies on the improvement of the wear resistance of titanium alloy hold great significance for improving its reliability and service life. The micro-groove structure has been proven to be useful for improving the lubrication performance during the wet sliding contact condition, thereby reducing the friction coefficient and wear [6]. Numerous fabrication technologies have been proposed for the machining of the micro-groove, including lithographic machining [7], micro electrical discharge machining [8], micro electrochemical machining [9] and micro mechanical machining [10,11,12]. The lithographic machining technology was more suitable for processing tiny nano-scale structures with a straight sidewall and high aspect ratio due to its high resolution and low removal rate [7]. The micro electrical discharge machining only could be used for the conductive material, and the feature structure was limited by the geometry size of the electrode tool. In addition, the micro electrochemical machining was difficult to obtain the high quality finished surface [9]. Notably, a considerable amount of investigations show that the micro mechanical machining is the most widely used, because it has a large dimension span and allows a high degree of freedom for the structural design as compared with other methods [11,13]. 

**Figure 1:**
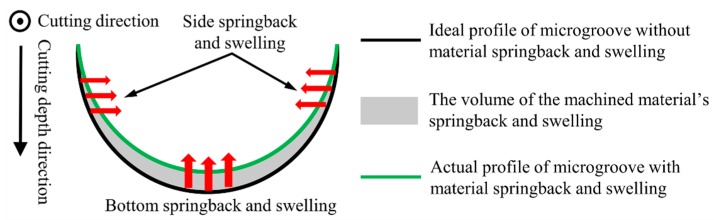
Schematic view of the material swelling and springback in the machining of micro-groove.

Titanium alloys have been considered to be typical hard-to-cut materials owing to their inherent properties such as the high chemical reactivity, high strain hardening (work hardening), low thermal conductivity and small deformation coefficient [14]. Hence, the high-quality and high-precision machining of the micro-groove on titanium alloy has always been a challenge with the conventional cutting (CC) process [15,16,17]. During the processing of a micro-groove on titanium alloy using the CC process, the generated cutting heat could not be effectively dissipated through the workpiece or chips because of the low thermal conductivity. Furthermore, the effect of the coolant was greatly limited because the coolant was vaporized before reaching the cutting zone [17]. Thus, the high cutting temperature and thermal stress exist in the narrow cutting area, which are sufficiently high to induce the plastic side flow of melted materials, and leaving the materials behind the cutting edge. Yip et al. [16] reported that the materials expand and their volume increases when the melted materials solidify again, especially at the bottom and side location of cutting edge. Therefore, there is an obvious deviation between the profile shape of the generated micro-groove and the ideal case due to the effect of the material swelling and springback, as shown in Figure 1. 

More remarkably, the effect of the material swelling and springback of titanium alloy was enhanced due to its low elastic modulus and thermal conductivity [16]. Furthermore, during the CC process, many surface defects such as adhered particles, a welded built-up edge (BUE) and plastic flow grooves appeared on the finished surface [15,18]. Previous studies have suggested that the high cutting temperature was the main factor that caused the formation of surface defect and the serious material swelling and springback [16,19]. Therefore, some coolant technologies, such as the coolant pressurized jet [20], cryogenic cooling [21], minimum quantity lubrication (MQL) [22] have been developed to reduce the cutting temperature. Further high-pressures increased the momentum of the coolant, which led to better heat transfers [17]. However, the environmental pollution and hazards to the operator occurred due to the high use of cutting fluids [23]. In the cryogenic cutting environment, the form accuracy of the machined part was difficult to guarantee and the hardness of workpiece was growing [24]. In MQL machining, a small amount of cooling/lubricating agents, as in the form of an aerosol, enter in the cutting zone for the advantage of effective cooling and lubrication at the tool–chip interface [22]. Krolczyk et al. [25] highlighted that this technology was a practical alternative to drying as well as flood cutting, which could reduce the use of cutting fluid and the manufacturing cost, thus achieving the eco-benign cutting environment. Maruda et al. [26,27] claimed that the MQL technology was performed better when the extreme pressure and anti-wear additives were added, which provided a significant improvement in the cutting tool wear rate. The MQL technology has been applied in many machining techniques, as well as in the wide range of workpieces (especially for Inconel alloys and titanium alloys) [28,29]. Nevertheless, the setup of MQL was complicated and troublesome [25]. Moreover, MQL technology required advanced equipment and caused a higher manufacturing cost, which limited its development [18]. Hence, a better machining method should be adopted to improve the machining performance of titanium alloy and reduce the level of material swelling and springback, thereby resulting in the high-quality and high-precision processing of micro-grooves on titanium alloy. 

The ultrasonic elliptical vibration assisted cutting (UEVC) is a promising cutting technique which shows particular advantages over CC, like lower machining forces, higher machining stability, less tool wear and a better surface finish [30,31,32]. Furthermore, the investigations of the fabrication of micro-groove structures assisted by the UEVC technology have gained more attention from researchers in recent years. Kim et al. [33,34] investigated the machining characteristics of micro-grooves on aluminum and brass by using the UEVC technology. Their results indicated that, in the UEVC process, the machining quality of micro-grooves was improved and the cutting forces were significantly decreased compared with the CC process. Suzuki et al [35,36] performed the micro-groove machining experiments on brittle materials by using the UEVC technology. The results showed that, due to the influence of the UEVC technology, the cutting forces reduced, the critical cutting depth increased and the machining accuracy of the micro-groove improved. Moreover, Zhang et al [11,12] compared and analyzed the machining characteristics of micro-grooves on the stainless steel (0Cr18Ni9) and brass by using the UEVC and CC processes. Their results demonstrated that the machining quality of micro-grooves improved and the cutting forces were reduced in the UEVC process, especially for stainless steel. Similarly, the experimental results obtained by Kurniawan et al. [37] indicated that the UEVC process has shown many advantages in the machining of micro-grooves on steel alloy compared to the CC process. 

Therefore, as discussed above, the UEVC process is effective to reduce the cutting forces, lower the surface roughness and attain better machining accuracy in micro-groove machining of easy-to-cut materials (such as copper, brass, and aluminum), brittle materials as well as steel materials. However, the UEVC technology is yet to be used in the machining of micro-grooves on titanium alloy. Moreover, a few studies have been conducted to assess the effect of material swelling and springback in micro-groove machining, which is the dominant factor to deteriorate the form accuracy and surface integrity of micro-grooves, especially for titanium alloy with the high level of material swelling and springback [16,38]. In this work, the UEVC technology is employed to investigate the machining characteristics of micro-grooves on Ti-6Al-4V alloy. In order to clarify the effective mechanisms of the UEVC technology in micro-groove machining of titanium alloy, comparative investigations on the surface defect, surface roughness, and material swelling and springback under the CC and UEVC processes are carried out. Moreover, the effects of different machining parameters on the machining quality of micro-grooves are compared with particular emphasis on the material swelling and springback. It is hoped that this work can provide a feasible method for high-quality and high-precision machining of micro-groove on titanium alloy.

## 2. Materials and Methods 

### 2.1. The UEVC Principle

**Figure 2:**
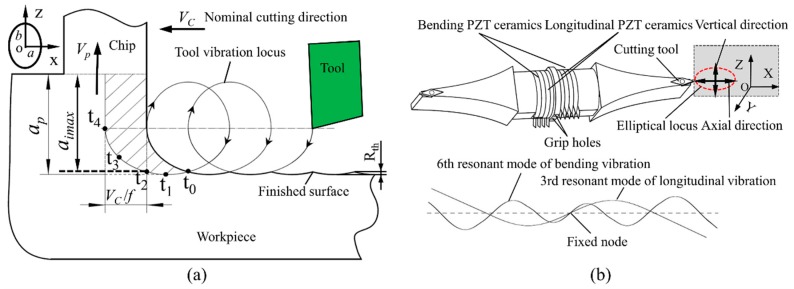
(**a**) Illustration of the ultrasonic elliptical vibration assisted cutting (UEVC) process, (**b**) Schematic diagram of the UEVC device.

Figure 2a shows the schematic illustration of the UEVC process, and Figure 2b presents the schematic diagram of the UEVC device used in this study. The UEVC device worked at the 3rd longitudinal resonant mode and the 6th bending resonant mode. Four groups of longitudinal and bending piezoelectric (PZT) ceramics were stacked between metal blocks. When the excitation signals applied to the longitudinal PZT ceramics and the bending PZT ceramics, the 3rd longitudinal and 6th bending resonant modes of the device were inspired, respectively. An elliptical vibration trajectory was obtained at the tool tip by combining the two resonant vibrations with some phase shift. The detailed working principle of the UEVC device was given in our previous work [39].

As shown in Figure 2a, the cutting tool elliptically vibrates in the *xoz* plane, which is formed by the nominal cutting direction (i.e., *x*-axis) and the chip flow direction (i.e., *z*-axis). Relative to the workpiece, the transient position of the cutting tool can be described as follows:

(1) $$x\left(t\right)=a\sin\left(2\pi ft\right)-V_{C}t,$$

(2) z(t)=bsin(2πft+θ).

Thus, the tool velocity relative to workpiece can be written as:

(3) $$x^{\prime }(t)=2\pi fa\cos(2\pi ft)-V_{C},$$

(4) $$z^{\prime }(t)=2\pi fb\cos(2\pi ft+\theta ).$$

where *a* and *b* are the vibration amplitudes in *x*-direction and *z*-direction, respectively. *θ* is the phase shift of the 3rd longitudinal resonant and 6th bending resonant, *V_C_* is the nominal cutting speed and *f* is the vibration frequency. In addition, it should be noted that the ratio of the nominal cutting speed to the peak horizontal vibration speed is an important parameter in the UEVC process. In this study, this ratio is named as TSR. And TSR can be written as:

(5) $$\mathrm{TSR}=\frac{V_{C}}{2\pi fa}.$$

It should be noted that the intermittent cutting only occurs when TSR < 1. Nath et al. have demonstrated that the machined surface quality improved with the decrease of the value of TSR, and the TSR was usually set to be less than one-twelfth in the ultra-precision machining process [40]. Hence, three different values of TSR, namely one of twelfth, one of twenty-four and one of forty eight, are selected by varying the normal cutting speed for studying the influence of TSR on the machining quality of micro-grooves on titanium alloy. It should be noted that different TSR values in the UEVC process can be implemented by adjusting the radius of the machining area. 

In the UEVC process, the three most important features are the intermittent cutting, the reduced instantaneous uncut chip thickness and the reversal of friction force between the tool and the chip [13]. As shown in Figure 2a, the cutting motion starts at time t_0_, and the cutting tool separates from workpiece at time t_4_. Thus, in each cycle of vibration, the contact time between the tool and workpiece is only (t_4_–t_0_). The cooling medium was more likely to enter the cutting area, and the cooling effect was enhanced [41]. As a result, the cutting temperature was reduced. As presented in Figure 2a, the instantaneous uncut chip thickness continuously varies, and is maximal at time t_2_. The maximal instantaneous uncut chip thickness (*a_imax_*) is also smaller than the nominal one (*a_p_*). This means that the cutting forces and cutting temperature during the UEVC process are smaller than in CC process. At time t_3_, the velocity of the cutting tool in the *z* direction is equal to the velocity of the chip flow. This can be represented by the following equation:

(6) $$z^{\prime }(t_{3})=2\pi fb\cos(2\pi ft_{3}+\theta )=V_{p}.$$

where *V_p_* is the velocity of the chip flow and is considered as a constant. According to the vibration equations of the tool (Equations (3) and (4)), the velocity of the tool in the z direction is increased in time period (t_4_–t_3_). Therefore, during the time period of (t_4_–t_3_), the friction force between the chip and tool is reversed. That means the friction force and the velocity of the chip flow have the same direction, which is conducive to break the chip and pull the chip away from the workpiece, and the results in a remarkable decrease in the cutting force, the suppression of the tool chatter, and an increase in the nominal shear angle [40,42]. In the UEVC process, the cutting forces and cutting temperature are reduced, the tool chatter is suppressed, and the chips are smoothly removed. Thus, the UEVC technology maybe a promising machining method to improve the machining performance of titanium alloy and reduce the level of material swelling and springback, thereby resulting in the high-quality processing of micro-grooves on titanium alloy.

### 2.2. Experimental Setup

**Figure 3:**
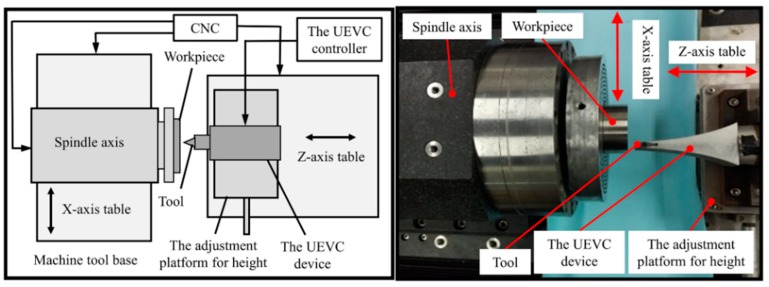
Diagram and picture of experimental setup.

In the experimental setup, a typical titanium alloy Ti-6Al-4V alloy, was chosen. The physical properties of Ti-6Al-4V alloy are listed in Table 1. The workpiece is held by the vacuum chuck attached on the spindle of the home-made ultra-precision machine tool, as shown in Figure 3. The machine tool mainly consists of an aerostatic spindle and two horizontal hydrostatic slideways. The UEVC device was positioned on the *z*-axis, and the high-precision adjustment platform was used to achieve the height adjustment of the UEVC device. It should be noted that the UEVC device could be considered as a traditional tool holder when it was not powered. The samples were round pie with the diameter of 50 mm and the height of 20 mm. The pre-turning was first completed with the CC process. Following this, a series of micro-groove machining experiments under different machining conditions were carried out. The experimental conditions and cutting parameters are listed in Table 2.

**Figure 4:**
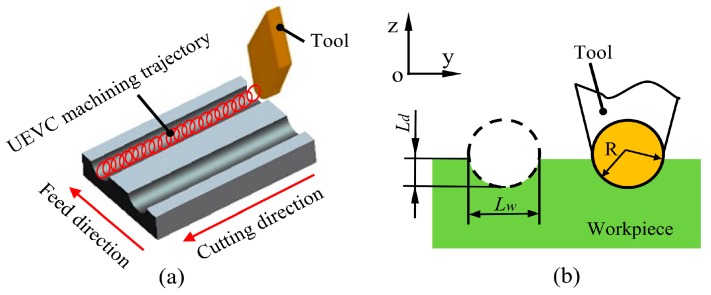
(**a**) Schematic view of the UEVC process in the machining of the micro-groove; (**b**) Enlarged view of the machined micro-groove.

Figure 4 displays the schematic view of the UEVC process in the machining of the micro-groove and the enlarged view of the generated micro-groove. In this study, the cutting tool with a round nose was used. In the ideal situation, the profile shape of the generated micro-groove is anticipated as same as the tool profile. The theoretical value of the micro-groove depth (*L_d_*) is equal to the predetermined depth of cut. The theoretical value of the micro-groove width is *L_w_*, and it can be expressed as:

(7) $$L_{w}=2\sqrt{R^{2}-{\left(R-L_{d}\right)}^{2}}$$

where R is the nose radius of the tool, and *L_d_* is the theoretical value of the micro-groove depth. Thus, the theoretical value of the micro-groove width can be obtained from Equation (7). 

## 3. Results and Discussion

### 3.1. Material Swelling and Springback

**Figure 5:**
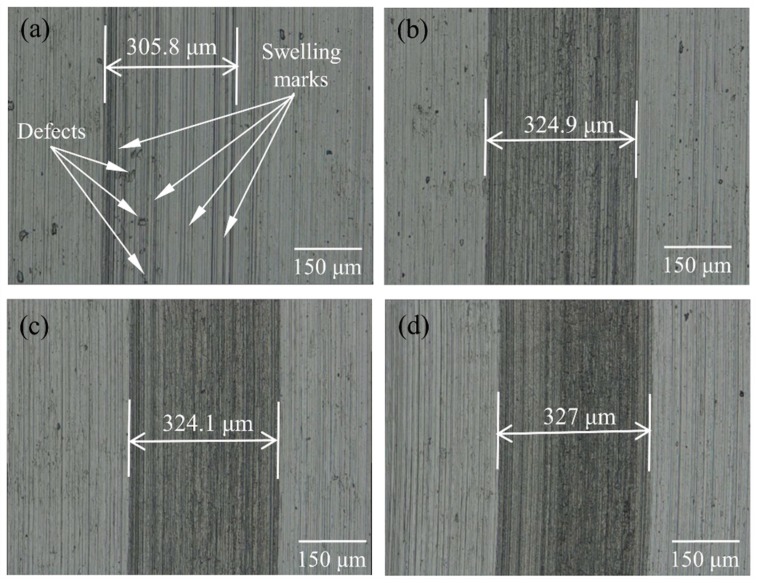
Micrographs of machined micro-groove (the predetermined depth of cut was 8 μm): (**a**) processed by CC, (**b**) processed by UEVC (TSR = 1/12), (**c**) processed by UEVC (TSR = 1/24) and (**d**) processed by UEVC (TSR = 1/48).

In the micro-groove machining of titanium alloy, the suppression of material swelling and springback is crucial. To evaluate whether the UEVC technology, especially at different TSR, meets to suppress the material swelling and springback, the machining quality assessment of the machined micro-grooves is respectively performed by ultra-depth 3D microscopy system (VHX 1000E; Keyence, Osaka, Japan) and a white light interferometer (NewView 5000; Zygo, Middlefield, CT, USA). The machining quality of the micro-grooves with the predetermined depth of cut of 8 μm produced by different processing methods was first analyzed. Figure 5 shows the micrographs of the generated micro-grooves. As shown in Figure 5a, the clear, obvious and straight swelling marks appeared on the bottom and the side of the micro-groove produced by the CC process. Numerous surface defects were randomly distributed on the finished surface. On the contrary, the machined surfaces of the micro-grooves were clear and smooth, with no surface defect and swelling marks under the UEVC process with different TSR, as shown in Figure 5b–d. These facts implied that the material swelling and springback effect was very obvious in the generated micro-groove produced by CC, and the material swelling and springback effect was obviously suppressed by using the UEVC technology. The underlying reasons could be explained by analyzing the basic function mechanisms of the UEVC technology. As discussed in Section 2.1, the intermittent cutting and the reduction of instantaneous cutting thickness existed in the UEVC process, which caused small cutting forces and resulted in little cutting heat. Furthermore, the reversed friction force led to the increase of the nominal shear angle, so the cutting forces and friction force could be further reduced. On the other hand, the intermittent cutting mechanism provided a more favorable heat dissipation condition. Thus, the cutting temperature in the UEVC process was far lower than the CC process, which resulted in the prominent reduction in the level of material swelling and springback during the processing of micro-grooves on titanium alloy. 

**Figure 6:**
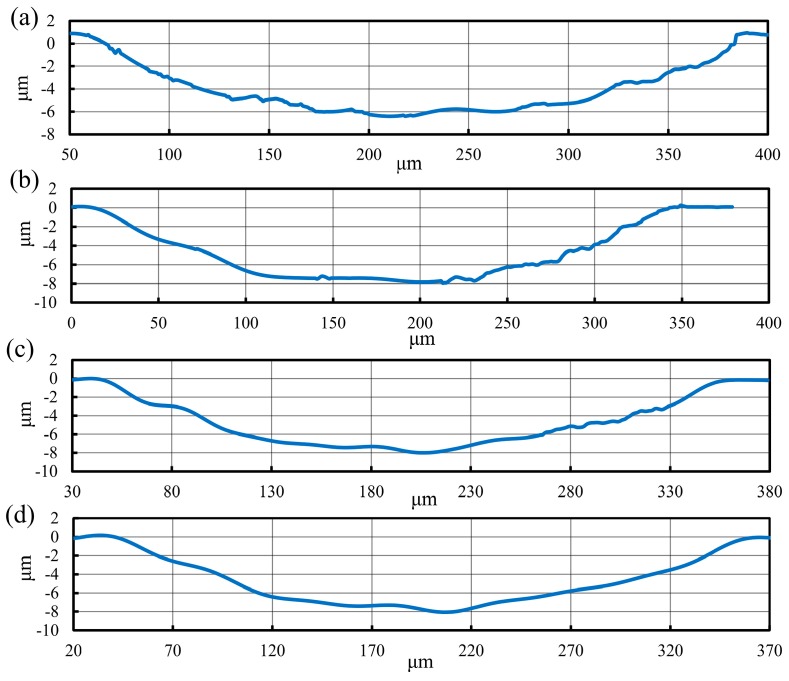
The cutting profiles of machined micro-groove (the predetermined depth of cut was 8 μm): (**a**) processed by CC, (**b**) processed by UEVC (TSR = 1/12), (**c**) processed by UEVC (TSR = 1/24) and (**d**) processed by UEVC (TSR = 1/48).

The reduction in the level of material swelling and springback was directly reflected in the dimensional parameters of the generated micro-groove. As displayed in Figure 5, the width of the micro-groove machined by the CC process was 305.8 μm, while the width of the micro-grooves machined by the UEVC process with different TSR were 324.9 μm (TSR = 1/12), 324.1 μm (TSR = 1/24) and 327 μm (TSR = 1/48), respectively. These implied that, during the processing of the micro-groove on titanium alloy using the CC process, the side swelling and springback effect was obvious and it introduced the larger volume of the material at the groove side, therefore, the width of generated micro-groove was smaller than the designed one (329.3 μm). During the processing of the micro-groove on titanium alloy using the UEVC process with different TSR values, the deviation values in the micro-groove width were small. The experimental results were all closed to the designed value. In order to further study the details of the generated micro-grooves, the cutting profiles were analyzed, as shown in Figure 6. Due to the effect of material swelling and springback, the ragged profile with wavy and vibration characteristics was obtained, as shown in Figure 6a. The depth of the micro-groove produced by the CC process was 6.4 μm, and it was much less than the designed value (8 μm), which indicated that a significant bottom swelling and springback appeared on the bottom surface of the generated micro-groove. Furthermore, the shape of the cutting profile was even distorted, and deviated largely from the tool shape. Thus, in the CC process, the actual parameters of the generated micro-groove greatly strayed from the designed parameters. In contrast, as shown in Figure 6b–d, the cutting profiles of the generated micro-grooves produced by UEVC process with different TSR displayed a smooth radius curve, and the depths were all close to 8 μm. It is worth noting that as the TSR value decreases, the cutting profile appears smoother. The cutting profile of micro-groove machined by the UEVC process with TSR = 1/48 has a few ripples and vibration marks.

**Figure 7:**
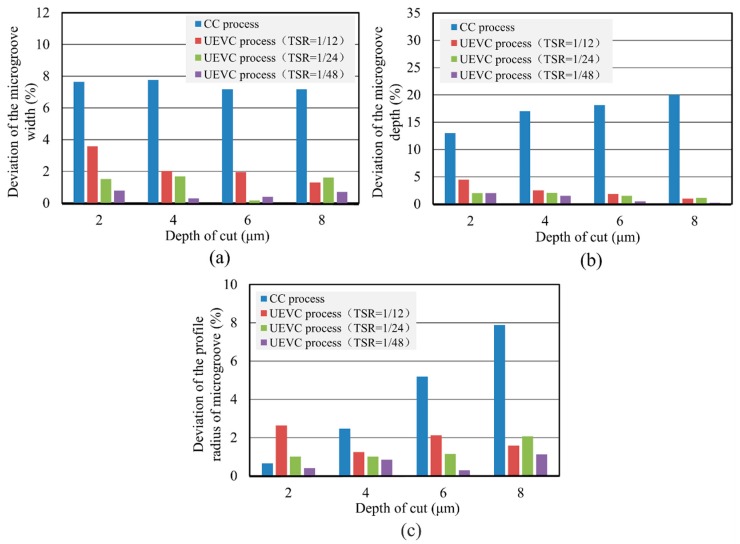
(**a**) Deviation of the micro-groove width, (**b**) deviation of the micro-groove depth, and (**c**) deviation of the profile radius of micro-groove.

For further investigating the effect of the predetermined depth of cut on material swelling and springback under different machining conditions, a series of experiments about micro-groove machining of titanium alloy were carried out. The measured parameters of the generated micro-grooves are presented in Table 3. In addition, according to the experimental data in Table 3, the deviations between the actual parameters of the generated micro-grooves and the theoretical values were analyzed. During the processing of the micro-groove on titanium alloy using the CC process, the deviations of the micro-groove width did not change significantly with the increase of the predetermined depth of cut, as shown in Figure 7a. Noting that, for all the predetermined depths of the cut, the deviations of the micro-groove width in the CC process were almost 7–8%, while the deviations of the micro-groove width in the UEVC process were smaller than 4%. In particular for the UEVC process with TSR = 1/48 and TSR = 1/24, the deviations of the micro-groove width were smaller than 2%. These facts indicate that the UEVC technology can effectively suppress the side swelling and springback during the micro-groove machining of titanium alloy, and in the UEVC process, TSR = 1/24 is recommended because the smaller TSR value means lower processing efficiency. 

Figure 7b presents the deviations of the micro-groove depth under the different processing conditions. In the CC process, the deviation value gradually increases with the increase of the predetermined depth of the cut. The deviation value reached 20%, when the predetermined depth of the cut was 8 μm. This can be explained by the fact that the increase of the predetermined depth of the cut led to an obvious increase in cutting forces and the friction force, thus, the cutting temperature was higher, which greatly increased the level of material swelling and springback. Conversely, the deviations of the micro-groove depth in the UEVC process were smaller than 5% for all predetermined depths of the cut, and were smaller than 2% when TSR = 1/48 or TSR = 1/24. Moreover, the deviations of the micro-groove depth were smaller with the decrease of TSR. This is because the decrease of TSR could further promote the superiority of the UEVC technology, that is, smaller cutting forces and a better heat dissipation condition could be obtained with smaller TSR. Moreover, it is worth noting that, with the increase of the predetermined depth of the cut, the suppression effect of the UEVC technology on material swelling and springback was more obvious. This happened maybe due to the following reasons. In the CC process, the larger depth of the cut means larger cutting forces and a higher cutting temperature. Thus, with the increase of the predetermined depth of the cut, the material swelling and springback becomes the dominant factor in the deviation of the micro-groove depth. However, as discussed above, the effect of material swelling and springback was effectively suppressed by using the UEVC technology. This means the dominant factor in the deviation of the micro-groove depth was eliminated. Therefore, the deviation of the micro-groove depth gradually decreased with the increase of the predetermined depth of the cut. 

As can be seen from Figure 7c, it is clear that, in the CC process, the deviation of the profile radius of the machined micro-groove rapidly increased with the increase of the predetermined depth of the cut. However, since the UEVC technology has a good suppression effect on the material swelling and springback in both of the side and bottom during the micro-groove machining of titanium alloy, the deviations of the profile radius of the micro-groove were small for all predetermined depths of the cut. It is worth noting that, for the UEVC process with TSR = 1/48 or TSR = 1/24, the deviations of the profile radius of the micro-groove were smaller than 2%. This indicated that the accuracy of the machined micro-groove could be ensured during the processing of the micro-groove on titanium alloy by using the UEVC process.

### 3.2. Surface Integrity

**Figure 8:**
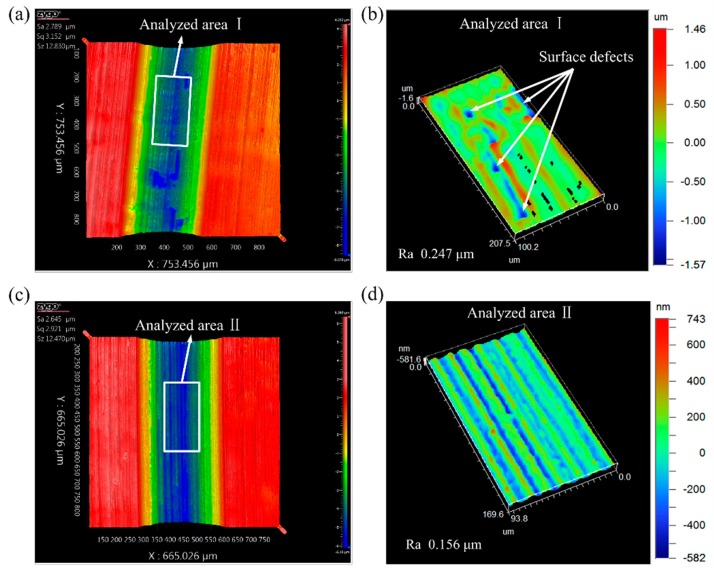

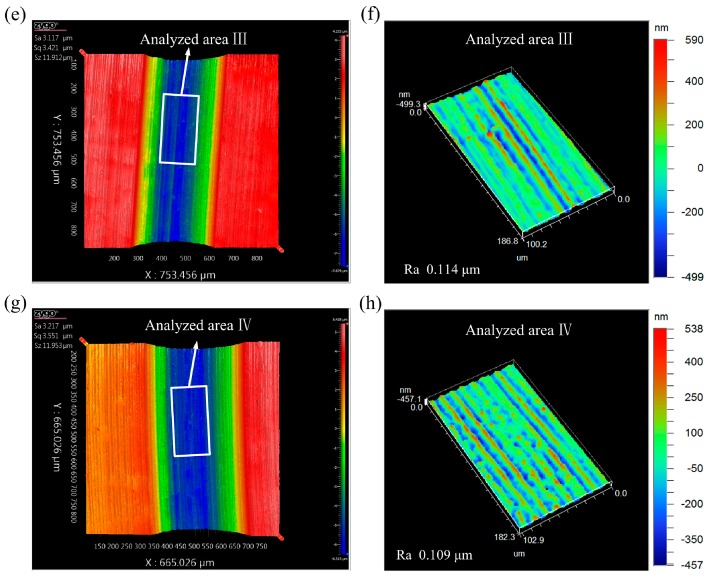
The views of machined micro-grooves (the predetermined depth of cut was 8 μm): (**a**) surface topography of machined micro-groove during CC; (**b**) surface roughness of analyzed area I; (**c**) surface topography of machined micro-groove during UEVC (TSR = 1/12); (**d**) surface roughness of analyzed area II; (**e**) surface topography of machined micro-groove during UEVC (TSR = 1/24); (**f**) surface roughness of analyzed area III; (**g**) surface topography of machined micro-groove during UEVC (TSR = 1/48); (**h**) surface roughness of analyzed area IV.

Surface integrity is an important indicator for evaluating the machined surface quality and has a large impact on the reliability and service life of the part. Figure 8 shows the surface topography views of the machined micro-grooves with the predetermined depth of cut of 8 μm. It can be observed from Figure 8a,b, that a series of surface defects including irregular ridges, cavities and tearing marks appeared on the micro-groove machined by using the CC process. This can be attributed to the following reasons. During the processing of the micro-groove on titanium alloy using the CC process, the high cutting temperature, large cutting forces and the existence of friction force, which elevated the softening degree of the workpiece material, induced the ruleless vibration of the cutting tool and promoted the formation of the built-up edge (BUE) [18]. The ruleless vibration of the cutting tool and the generated BUE promoted the formation of cavities and tearing marks. In addition, as discussed in Section 3.1, the level of material swelling and springback was very high in the CC process. The irregular ridges were generated by the combined effect of the high level of material swelling and springback and the ruleless vibration of the cutting tool. It should be noted that these defects vastly degraded the surface integrity and the accuracy of the generated micro-groove. 

In contrast, the machined micro-grooves produced by the UEVC process displayed nearly no surface defects on the bottom and side surfaces, as shown in Figure 8c–h. This can be attributed to the following two reasons. The first reason is that, compared to the CC process, the cutting forces and friction force were small due to the features of the UEVC technology such as the intermittent cutting, the reduction of instantaneous cutting thickness and the reversal of the friction force. Additionally, in the UEVC process, a favorable heat dissipation condition was provided because of its intermittent cutting nature. Consequently, the cutting temperature in the UEVC process was far lower than in the CC process, thereby resulting in the effective suppression for the ruleless vibration of the cutting tool and the formation of BUE. The second reason is that, the material swelling and springback was successfully suppressed during the processing of the micro-groove on titanium alloy using the UEVC process, as discussed in Section 3.1. Hence, there were almost no surface defects on the machined surface, as shown in Figure 8d,f,h. Simultaneously, it can be seen that some small and straight ridges also appeared on the machined micro-grooves produced by the UEVC process, especially for TSR = 1/12. This may be caused by the micro notches appearing on the cutting edge of the tool. The effect of the micro notches on the machined surface quality decreased with the decrease of TSR. The reason is that the smaller TSR means the smaller cutting forces and instantaneous uncut chip thickness. As shown in Figure 8f,h, the bottom surface roughness of the machined micro-groove produced by the UEVC process with TSR = 1/24 and TSR = 1/48 were 0.114 μm and 0.109 μm, respectively. It should be noted that the size of the analysis area is approximately 100 μm × 180 μm. However, the bottom surface roughness of the machined micro-groove produced by the CC process was 0.247 μm, which was more than two times than the roughness in the UEVC process with TSR = 1/24 or TSR = 1/48. Therefore, the application of the UEVC technology led to an obvious improvement of the machined micro-groove in surface integrity, which was consistent with the experimental result from the research literature [33,34,35,36,37].

**Figure 9:**
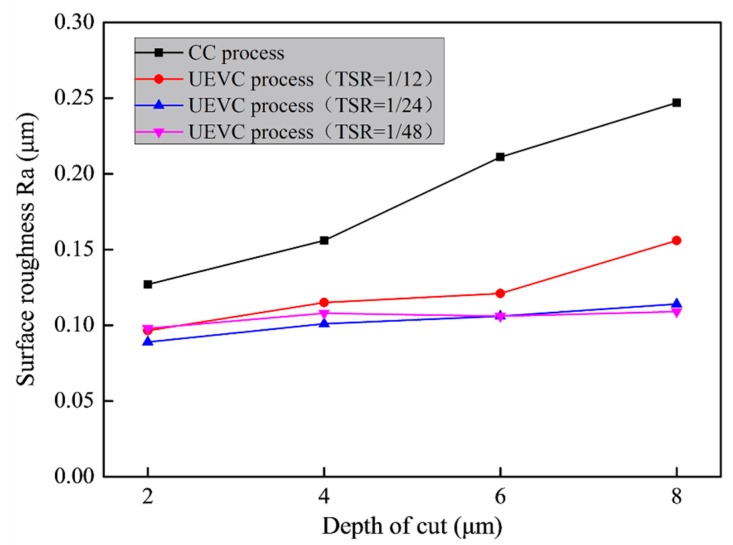
Influence of the predetermined depth of cut on the bottom surfaces roughness of the machined micro-grooves produced by different processing conditions.

Figure 9 presents the bottom surface roughness values of the machined micro-grooves produced by different processing conditions. For each generated micro-groove, the bottom surface roughness was tested five times and the recorded surface roughness was the average value. The measurement area size was approximately 100 μm × 180 μm. It can be seen that the surface roughness value increased quickly with the increase of the predetermined depth of the cut during the CC process. As discussed above, many surface defects including irregular ridges, cavities and tearing marks appeared on the machined micro-groove under the CC process. It is obvious that a bigger depth of the cut means larger cutting forces and friction force, thereby resulting in the higher cutting temperature. The higher cutting temperature caused more serious ruleless vibration of the tool and the material swelling and springback. Thus, it can be inferred that more machining defects appeared on the generated micro-groove with the increase of the predetermined depth of the cut. The appearance of above-observed machining defects lower the surface quality of the generated micro-groove. However, the surface roughness values did not obviously change with the increase of the predetermined depth of the cut during the UEVC process with TSR = 1/24 or TSR = 1/48. The roughness values were approximately 0.1 μm. These results were consistent with the previous discussion which stated that a significant suppression of the formation of the surface defect was due to the use of the UEVC technology. The experimental results certified that the reduction of material swelling and springback and the improvement of surface integrity could be achieved simultaneously by using the UEVC technology.

## 4. Conclusions

In this work, the UEVC technology is firstly introduced into the micro-groove machining of Ti-6Al-4V alloy to improve the machining performance and reduce the level of material swelling and springback, thereby realizing the high-quality and high-precision processing of micro-groove. Based on the theoretical analysis and the experiment results, the main conclusions can be drawn as follows:

1. During the processing of the micro-groove on titanium alloy using the CC process, the clear, obvious and straight swelling marks appeared on the bottom and the side of the generated micro-groove. These facts implied that the material swelling and springback effect was very obvious. Further, with the increase of the predetermined depth of the cut, the deviation of the micro-groove width did not change significantly, while the deviation of the micro-groove depth gradually increased. Moreover, the deviation of the profile radius of the micro-groove rapidly increased with the increase of the predetermined depth of the cut. Remarkably, the profile shape of the generated micro-groove was distorted, and deviated largely from the tool shape. Thus, the actual parameters of the micro-groove machined by the CC process greatly strayed from the designed parameters.
2. During the processing of the micro-groove on titanium alloy using the UEVC process, no surface defect and no swelling mark appeared on the machined surfaces of the generated micro-grooves. The profile shape of the generated micro-grooves was smooth, and the profile became smoother with the decrease of TSR. This indicated that the material swelling and springback effect was obviously suppressed. As the predetermined depth of the cut increased, the deviations between the designed parameters and experimental results of the micro-groove width, depth and the profile radius did not significantly change. It is noticeable that, for all TSR, the percentage errors of the generated micro-groove parameters were smaller than 5%, and were smaller than 2% when TSR = 1/48 or TSR = 1/24. This indicated that the accuracy of the machined micro-groove improved significantly by using the UEVC technology.
3. A series of surface defects including irregular ridges, cavities and tearing marks appeared on the micro-groove machined by using the CC process, and these defects vastly degraded the surface integrity and the accuracy of the generated micro-groove. In contrast, the generated micro-grooves produced by the UEVC process displayed nearly no surface defects. Moreover, the bottom surface roughness of the machined micro-grooves increased quickly with the increase of the predetermined depth of the cut during the CC process. However, the bottom surfaces roughness of the machined micro-grooves did not obviously change with the increase of the predetermined depth of the cut during the UEVC process. The roughness values were approximately 0.1 μm, when TSR = 1/48 or TSR = 1/24. Therefore, the application of the UEVC technology led to an obvious improvement of the machined micro-groove in surface integrity.
4. The experimental results certified that the reduction of the material swelling and springback and the improvement of surface integrity can be achieved simultaneously by using the UEVC technology. The value of TSR has an obvious effect on the action mechanism of the UEVC technology, and the decrease of TSR can further promote the superiority of the UEVC technology. In this study, TSR = 1/24 is recommended. Further investigation should be made to obtain the preferable machined surface by optimizing the combination of processing parameters with the consideration of tool wear.

## Figures and Tables

**Table 1:** Physical properties of Ti-6Al-4V alloy.

Tensile Strength (MPa)	Elastic Modulus (GPa)	Hardness (HRC)	Density kg/m^3^	Specific Heat Capacity (J∙kg^−1^∙°C^−1^)	Thermal Conductivity (W∙m^−1^∙°C^−1^)
902	16	32	4500	610	7.6

**Table 2:** Experimental parameters.

Processing Method		CC Process				UEVC Process			
Vibration parameters	Frequency (kHz)	-				29.75			
	Amplitude in cutting direction (μm)	-				6			
	Amplitude in cutting depth direction (μm)	-				4			
	Phase shift (°)	-				150			
Cutting parameters	Spindle speed (r/min)	480				20			
	Feed rate (μm/r)	500				500			
	Depth of cut (μm)	2,	4,	6,	8	2,	4,	6,	8
Cutting Tool	Material	Carbide							
	Rake angle (°)	0							
	Clearance angle (°)	11							
	Nose radius (mm)	1.698							
Workpiece	Material	Ti-6Al-4V alloy							
	Dimension (mm)	*Φ*50 × *L*20							
Cutting fluid		No							

**Table 3:** The designed parameters and experimental results of the machined micro-grooves.

Test No.	Parameters of the Micro-Groove	Designed Values	Results of CC Process	Results of UEVC Process (TSR = 1/12)	Results of UEVC Process (TSR = 1/24)	Results of UEVC Process (TSR = 1/48)
1	Micro-groove depth	2	1.74	1.91	1.96	1.96
	Micro-groove width	164.8	152.2	158.9	162.3	163.5
	Profile radius	1698	1709	1653.4	1681	1704.9
2	Micro-groove depth	4	3.32	3.89	3.91	3.94
	Micro-groove width	233	214.9	228.3	229	232.3
	Profile radius	1698	1740.4	1676.8	1678.5	1712.5
3	Micro-groove depth	6	4.91	5.89	5.91	5.97
	Micro-groove width	285.2	264.7	279.6	284.7	284.1
	Profile radius	1698	1786.2	1662	1717.3	1693
4	Micro-groove depth	8	6.4	7.92	7.91	7.98
	Micro-groove width	329.3	305.8	324.9	324.1	327
	Profile radius	1698	1829.7	1670	1663.9	1678.9

## Nomenclature

UEVC	ultrasonic elliptical vibration assisted cutting
TSR	the ratio of the nominal cutting speed to the peak horizontal vibration speed
*a*	the vibration amplitudes in x-direction
*b*	the vibration amplitudes in z-direction
*a_imax_*	the maximal instantaneous uncut chip thickness
*V_p_*	the velocity of the chip flow
*L_w_*	the theoretical value of the micro-groove width
CC	conventional cutting
PZT	piezoelectric
*Θ*	the phase shift of the 3rd longitudinal resonant and 6th bending resonant
*V_C_*	the nominal cutting speed
*F*	the vibration frequency
*a_p_*	the nominal instantaneous uncut chip thickness
*L_d_*	the theoretical value of the micro-groove depth
R	the nose radius of the tool
